# Molecular Signatures of the Primitive Prostate Stem Cell Niche Reveal Novel Mesenchymal-Epithelial Signaling Pathways

**DOI:** 10.1371/journal.pone.0013024

**Published:** 2010-09-30

**Authors:** Roy Blum, Rashmi Gupta, Patricia E. Burger, Christopher S. Ontiveros, Sarah N. Salm, Xiaozhong Xiong, Alexander Kamb, Holger Wesche, Lisa Marshall, Gene Cutler, Xiangyun Wang, Jiri Zavadil, David Moscatelli, E. Lynette Wilson

**Affiliations:** 1 Department of Cell Biology, New York University School of Medicine, New York, New York, United States of America; 2 Department of Pathology, New York University School of Medicine, New York, New York, United States of America; 3 Division of Immunology, University of Cape Town, Cape Town, South Africa; 4 Department of Science, Borough of Manhattan Community College/City University of New York, New York, New York, United States of America; 5 Department of Urology, New York University School of Medicine, New York, New York, United States of America; 6 Amgen Inc, South San Francisco, California, United States of America; 7 Pfizer Inc, Groton, Connecticut, United States of America; 8 NYU Cancer Institute, New York University School of Medicine, New York, New York, United States of America; 9 Center for Health Informatics and Bioinformatics, New York University Medical Center, New York, New York, United States of America; University of São Paulo, Brazil

## Abstract

**Background:**

Signals between stem cells and stroma are important in establishing the stem cell niche. However, very little is known about the regulation of any mammalian stem cell niche as pure isolates of stem cells and their adjacent mesenchyme are not readily available. The prostate offers a unique model to study signals between stem cells and their adjacent stroma as in the embryonic prostate stem cell niche, the urogenital sinus mesenchyme is easily separated from the epithelial stem cells. Here we investigate the distinctive molecular signals of these two stem cell compartments in a mammalian system.

**Methodology/Principal Findings:**

We isolated fetal murine urogenital sinus epithelium and urogenital sinus mesenchyme and determined their differentially expressed genes. To distinguish transcripts that are shared by other developing epithelial/mesenchymal compartments from those that pertain to the prostate stem cell niche, we also determined the global gene expression of epidermis and dermis of the same embryos. Our analysis indicates that several of the key transcriptional components that are predicted to be active in the embryonic prostate stem cell niche regulate processes such as self-renewal (e.g., E2f and Ap2), lipid metabolism (e.g., Srebp1) and cell migration (e.g., Areb6 and Rreb1). Several of the enriched promoter binding motifs are shared between the prostate epithelial/mesenchymal compartments and their epidermis/dermis counterparts, indicating their likely relevance in epithelial/mesenchymal signaling in primitive cellular compartments. Based on differential gene expression we also defined ligand-receptor interactions that may be part of the molecular interplay of the embryonic prostate stem cell niche.

**Conclusions/Significance:**

We provide a comprehensive description of the transcriptional program of the major regulators that are likely to control the cellular interactions in the embryonic prostatic stem cell niche, many of which may be common to mammalian niches in general. This study provides a comprehensive source for further studies of mesenchymal/epithelial interactions in the prostate stem cell niche. The elucidation of pathways in the normal primitive niche may provide greater insight into mechanisms subverted during abnormal proliferative and oncogenic processes. Understanding these events may result in the development of specific targeted therapies for prostatic diseases such as benign prostatic hypertrophy and carcinomas.

## Introduction

Stem cells reside within restricted microenvironments known as niches. Quiescence, self-renewal and differentiation of stem cells are regulated by intrinsic and extrinsic mechanisms. Intrinsic mechanisms include those that alter the epigenetic state of these cells such as those regulated by chromatin modifiers like the polycomb group proteins. Extrinsic mechanisms comprise those regulated by the niche and include direct interactions between stem cells and their supporting mesenchyme mediated by integrins and cadherins as well as locally secreted and membrane-bound growth factors. Elucidating the important signals in stem cell niches may delineate the mechanisms involved in regulating stem cell self-renewal and the maintenance of their multipotentiality. The best characterized mammalian stem cell niches are the epidermal niche in the bulge region of the hair follicle [1], the intestinal epithelial niche [2], the hematopoietic stem cell niche in the bone marrow [3] and the neuronal stem cell niche [4]. Although these niches encompass a wide variety of cells and tissues they share a number of common properties. First, each niche contains a supportive stromal element that may differ depending on the niche. For example endothelial cells nurture the neuronal niche while N-cadehrin-positive osteoblastic lining cells support the hematopoietic stem cell niche. Second, the niche provides physical contact and anchoring that is necessary for stem cell self-renewal. Adhesion molecules such integrins and E- and N-cadherin anchor stem cells to the extracellular matrix. Third, extrinsic factors that control the number and fate of the stem cell population are generated within the niche. These include various signaling molecules such as the Wnts, bone morphogenic proteins (BMPs), fibroblast growth factors (FGFs), Notch and Shh that control, positively and negatively, the self-renewal and commitment of stem cells. An important function of the niche is to regulate precisely the balance between self-renewal and differentiation in order to maintain optimal organ function at all times. The aberrant proliferation of stem cells within the niche may result in tumorigenesis. Therefore, one of the most important functions of the niche is its ability to regulate the balance between stem cell self renewal and quiescence [5].

In the adult animal the prostatic stem cell niche resides in the proximal region of murine prostatic ducts [6], [7], [8]. Interestingly, this is also the site of origin of prostate tumors [9], indicating that these may arise in stem cells. Although prostatic epithelial stem cells can be isolated from this region based on their high expression of Sca-1 [6], [10], their corresponding adjacent mesenchyme cannot be isolated due to lack of identifiable markers. In order to gain insights into the mechanisms that regulate the stem cell niche in the prostate we isolated epithelial cells (urogenital sinus epithelium, UGE) and their adjacent mesenchyme (urogenital sinus mesenchyme, UGM) from the embryological niche (urogenital sinus, UGS) from which the prostate develops [11]. To our knowledge this is the only mammalian stem cell niche from which pure samples of both the epithelial stem cells and their surrounding mesenchyme can readily be obtained.

The prostatic mesenchyme has a significant role in regulating the growth and behavior of the adjacent prostatic epithelium [7], [11]. Indeed, perturbations in growth factor production by the mesenchyme can induce tumors in the adjacent normal epithelium [12], [13]. Many of the epithelial/mesenchymal interactions uncovered in the embryonic prostatic niche are likely to be maintained in the adult niche. Perturbations in these signals may result in benign prostatic hyperplasia and prostate carcinomas as both of these diseases are thought to arise from aberrant stem cell proliferation. Understanding the interactions in the prostate stem cell niche may therefore result in the development of specific targeted therapies for these two common prostatic diseases.

In this study we generated a compendium of transcripts that are differentially expressed in embryonic UGE versus UGM. We further utilized computational gene expression analysis to decipher the potential transcriptional factors (TFs) and the main biological functionalities that are predicted to characterize the embryonic prostatic stem cell niche. The transcriptional components that are likely to be active in the embryonic prostate stem cell niche may regulate processes such as self-renewal (e.g., E2f and Ap2), lipid metabolism (e.g., Srebp1) and cell migration (e.g., Areb6 and Rreb1). In addition, based on their differential expression levels, we offer several ligand-receptor interactions that may be relevant in controlling signals in the stem cell niche including the Wnt/β-catenin, ephrin, Notch, Sonic hedgehog (Shh), FGF, TGF-β and bone morphogenic protein signaling pathways.

## Materials and Methods

### Cell preparation

#### Isolation of cell populations

All animal care and procedures were performed in compliance with New York University institutional review board requirements. UGE and UGM were isolated from the urogenital sinus of 16-day-old C57BL/6 murine embryos after digestion with trypsin [14] and added to TRIzol. Epidermis and dermis were prepared from skin removed from the back of E16 mouse embryos. Skin was incubated overnight at 4°C in 2 ml of Dispase solution (Gibco, Grand Island, NY, Cat. No, 17105-041, 0.4 mg/ml) in DMEM containing 10% FCS [15], [16]. The dermis and epidermis were separated manually using a dissecting microscope and added to TRIzol.

### Real-Time PCR

One µg of total RNA was reverse-transcribed at 52° C for 1 hour using the Thermoscript RT-PCR system (Invitrogen, Carlsbad, CA). 20 ng of resultant cDNA was used in a Q-PCR reaction using an iCycler (Biorad, Hercules, CA) and pre-designed TaqMan Gene Expression Assays (Applied Biosystems, Foster City, CA). Cycle threshold values for RNA samples obtained from triplicate cell samples were averaged; amounts of target were interpolated from standard curves and normalized to the housekeeping genes *Hprt* and *Tbp*.

### RNA isolation and microarray hybridization

We established transcriptional profiles for UGE, UGM, epidermis and dermis. Six replicates of UGE, five replicates of UGM, four replicates of epidermis and four replicates of dermis samples were analyzed. RNA was isolated by chloroform extraction and precipitated using isopropanol. Additional column purification was conducted (RNeasy mini kit, Qiagen, Valencia, CA) and the resulting RNA treated to remove DNA contamination (DNAfree, Ambion, Austin, TX). RNA concentration was assessed spectrophotometrically using a NanoDrop® ND-1000 Spectrophotometer (Wilmington, DE). Protocols produced clean RNA with OD 260/280>1.7. RNA quality was assessed using an Agilent 2100 Bioanalyzer (Agilent Technologies, Santa Clara, CA). Samples with a RNA Integrity Number (RIN) >7.0 were considered suitable for labeling and 20 ng were labeled using the GeneChip two-cycle target labeling kit (Affymetrix, Santa Clara, CA). Ten micrograms of labeled and fragmented cRNA were then hybridized at 45°C for 16 h to the mouse genome MOE430 2.0 array (Affymetrix Santa Clara, CA) which interrogates ∼45,000 transcripts. Raw expression data were analyzed using GCOS 1.4 (Affymetrix, Santa Clara, CA). Data were normalized to a target intensity of 500 to account for differences in global chip intensity. The data discussed in this publication have been deposited in NCBI's Gene Expression Omnibus and are accessible through GEO Series accession number GSE17797 (http://www.ncbi.nlm.nih.gov/geo/query/acc.cgi?acc=GSE17797).

### Analysis of functional categories

We utilized functional annotations of murine genes provided by the Murine Genome Informatics, which uses the standard vocabulary introduced by the Gene Ontology (GO) consortium. Enriched functional categories (*p*≤0.01, after correction for multiple testing) were identified in each of the gene sets using EXPANDER, in which hypergeometric calculation is used to determine over-represented GO functional categories in a target set relative to a background set (the entire collection of putative murine genes) [17]. To avoid biases, genes represented by multiple probe sets were counted only once.

### Computational analysis of promoter cis-regulatory elements

For promoter analysis we applied EXPANDER [17] to detect cis-regulatory promoter elements that control the observed transcriptional alterations in the gene expression clusters. Given target and background sets of promoters, EXPANDER performs statistical tests to identify TFs whose binding-site signatures are significantly over-represented in the target set relative to background (TF enrichment is indicated by *p*-value) [17]. Both strands of each promoter were scanned for putative binding sites (spanning the transcriptional start site from 1000 bp upstream to 200 bp downstream). The enrichments identified in this study were robust, as they remained stable over a large range of threshold values.

## Results and Discussion

### Isolation of urogenital sinus epithelium and its mesenchyme

To identify the variations in gene transcripts in embryonic UGE versus UGM we separated UGE and UGM cell populations from 16-day-old murine embryos and isolated RNA from these cells. In order to distinguish genes that are shared by other developing epithelial/mesenchymal compartments in the embryo from those that pertain to the prostate stem cell niche, we also isolated RNA from the epidermis and dermis of the same embryos. For each cell population we had at least 4 repetitive samples (each sample was generated by pooling tissue from 20 embryos for UGE/UGM samples and from 15 embryos for epidermis/dermis samples). The isolated RNA was processed and prepared for hybridization to microarrays and analyzed as described in Materials and Methods.

**Figure 1:**
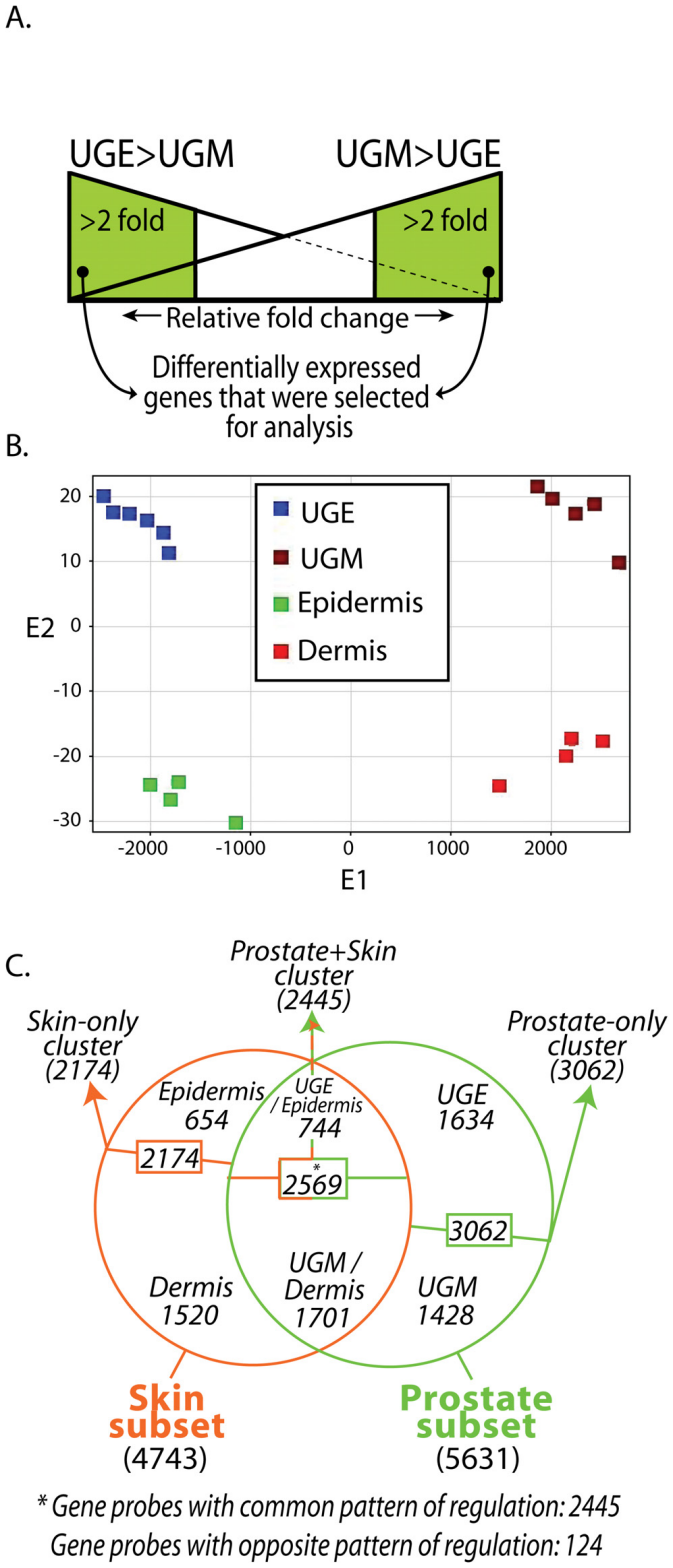
Definition of gene sets that are differentially expressed in the prostate stem cell niche. A. Genes that were differentially expressed by more than 2-fold in each compartment versus the other were selected for analysis of enriched functional categories, TF-binding motifs and ligand-receptor interactions. The line sloping down represents UGE vs UGM expression levels and the line sloping up represents UGM vs UGE expression levels. B. A plot of the principal components analysis (PCA) of differentially expressed gene sets derived from epithelial and mesenchymal compartments of the prostate and the skin. Expression data from the “*valid genes*” used in our analysis were subjected to PCA mapping. The positions of the samples with respect to the first two Eigenvectors (E1 and E2) are shown. The pattern is indicative of good separation of the four populations and good reproducibility within the replicate samples of each group. Each group is designated by a different color. C. Venn diagram detailing the number of transcripts (gene probes) shared and distinct among prostate and skin subsets. The numbers of transcripts within each subset is given in brackets outside the Venn chart (Prostate-subset (5631 transcripts); Skin-subset (4743 transcripts)). The numbers of transcripts within each cluster (*Prostate-only*, *Prostate+Skin* and *Skin-only*) are given in Italics adjacent to each arrowhead. The number of transcripts within the epithelial and mesenchymal compartments of each cluster (i.e., the gene sets) are depicted inside the slices of the Venn chart.

To reveal the genes that encode signaling molecules that may participate in cellular interactions between these two compartments in the prostate stem cell niche, we based our analysis on the genes that are differentially expressed between the UGE and the UGM. These genes reflect those that are distinctively overexpressed by one of the compartments relative to the other. Defining these differentially expressed gene transcripts may identify the unique components that are provided by each of the compartments in the niche. It is important to note that the genes that have similar expression levels in these two compartments (i.e., not differentially expressed) are omitted from our analysis. It is possible that these genes may encode regulators that contribute to signaling in the niche. We however focused our observations on the differentially expressed genes as we consider that the primary regulatory environment in the niche is likely to be due to specific molecules contributed by either one compartment or the other (**Figure 1A**).

### A. Analysis of gene expression and definition of gene sets

Utilizing ArrayAssist (Stratagene, La Jolla, CA), raw Affymetrix CEL files were processed by applying the MAS5 algorithm, to assign detection calls (Present/Marginal/Absent) for each probe set that were subsequently used in downstream data filtering. To generate normalized expression levels, we used PLIER (probe logarithmic intensity error) algorithm, a model-based, multi-array signal estimator which produces more accurate probe-set signal values [18]. The combination of these metrics was used for data filtering to obtain 34,087 “*valid genes*”, representing transcripts (gene probes) with signals >20 and detected as Present in at least one sample. A principle components analysis (PCA) (mean centered) calculated over the entire list of *valid genes* by ArrayAssist, shows an appropriate reproducibility and separation among the different types of cells (**Figure 1B**). Notably, the first principal component, that represents the greatest fraction of the variability in the gene expression, separates the prostate-derived cells from the skin-derived cells, while the second principal component, representing the second greatest amount of information in the expression data, discriminates between UGE/epidermis and UGM/dermis samples. This indicates significant differences in gene expression patterns between the tissue types. Thus the patterns of the two epithelial tissues (UGE and epidermis) and the two mesenchymal tissues (UGM and dermis) confirm similarities in gene expression patterns between tissues derived from a related ontogeny.

To define genes that were altered in a statistically significant manner, the samples were grouped according to their site of origin (UGE and UGM; epidermis and dermis), intensities of the *valid gene* transcripts were log2-transformed and subjected to further statistical analysis utilizing an unpaired t-test (Benjamini-Hochberg corrected *p*<0.01) between the UGE and the UGM samples and between the epidermis and the dermis samples. Two gene subsets representing the genes that are differentially expressed between the epithelial and the mesenchymal compartments were generated:

1. a “prostate” subset containing 5,631 transcripts whose expression was altered (up or down-regulated >2-fold) between the UGE and UGM samples and
2. a “skin” subset containing 4743 transcripts whose expression was altered (up or down-regulated >2-fold) between the epidermis and dermis samples (**Figure 1C**, **Tables S1**, **S2**).

We determined if we could discern common expression patterns shared between these two subsets and patterns that were unique to the prostate stem cell niche subset. Intersection of the two subsets resulted in the generation of three clusters (*Prostate-only*, *Prostate+Skin* and *Skin-only*) of mutually or exclusively expressed genes (**Figure 1C**). A distinct transcriptional expression pattern derived from the prostate stem cell niche cell population is manifest by 3,062 transcripts that are exclusively altered in the prostate stem cell niche (*Prostate-only* cluster). A smaller distinct pattern was evident in the skin cell populations represented by 2,174 transcripts (*Skin-only* cluster). This implies that the prostate niche has a greater number of genes that are differentially expressed between the epithelial and the mesenchymal compartments than noted in the skin compartment. This indicates that the unique transcriptional program that regulates the cellular signaling of the prostate stem cell niche may be more complex than the transcriptional program that regulates the molecular interactions in the epidermis/dermis. Interestingly, within the cluster that overlaps between the prostate stem cell niche and the epidermis/dermis subsets, the *Prostate+Skin* cluster, we found a substantial number of transcripts (2,569) whose differential expression is altered in both the prostate and epidermis/dermis subsets (**Figure 1C**). These data indicate that approximately half of the differentially expressed transcripts of the prostate and skin epithelial/mesenchymal subsets are shared while the remaining half represent a unique group of transcripts whose gene expression is specifically altered either in the prostate or in the skin.

To further analyze global transcriptional patterns that pertain to primitive prostate and skin epithelial/mesenchymal subsets, we determined the epithelial and the mesenchymal transcripts that are up-regulated in each of the three clusters. In the *Prostate-only* cluster we identified 1,634 transcripts (*Prostate-only UGE* gene set) that are differentially overexpressed in the UGE relative to the UGM (>2-fold) and 1,428 transcripts (*Prostate-only UGM* gene set) that are differentially up-regulated in the UGM over the UGE. In the *Skin-only* cluster we identified 654 transcripts (*Skin-only epidermis* gene set) that are differentially increased in the epithelium over the dermis and 1,520 transcripts (*Skin-only dermis* gene set) that are differentially up-regulated in the dermis over the epidermis (**Figure 1**, **Table S3**). In the common cluster (*Prostate+Skin* cluster, which contains 2,569 common altered transcripts) our analysis identified 2,445 transcripts that have a common regulation pattern shared by both the epithelial compartment and the mesenchymal compartment of both the prostate and skin epithelial/mesenchymal subsets; 744 transcripts of this cluster were up-regulated in both the UGE and the epidermis (Prostate+*Skin UGE/epidermis* gene set) relative to the UGM or the dermis, whereas 1,701 transcripts were up-regulated in both the UGM and the dermis (Prostate+*Skin UGM/dermis* gene set) relative to the UGE or the epidermis (**Figure 1**, **Table S3**). This indicates that the majority of the genes of the *Prostate+Skin* cluster (2445/2569; >95%) share a common regulatory pattern within their respective epithelial or mesenchymal compartments.

### B. Functional and promoter sequence analysis of gene expression in the prostate stem cell niche

By analyzing the *Prostate-only* cluster and the *Prostate+Skin* and *Skin-only* clusters we were able to determine attributes that are unique to prostate and those that are shared by both prostate and skin. The *Prostate-only* cluster reflects a unique cohort of genes that are important for regulating the dynamics and interactions in the prostate stem cell niche. Analysis of the two gene sets of this cluster, *Prostate-only UGE* and *Prostate-only UGM* gene sets, indicates commonalities in functional categories and promoter TF-binding motifs between these two gene sets (**Table 1**, **Tables S4**, **S5**). Functional categories which reflect expected features of stem cell biology are *anatomical structure development* and *cell adhesion* [19] and *epidermis development*, *ion channel activity* and *calcium ion binding* [20], [21]. The unique cohort of genes that are represented exclusively in the epidermis/dermis (*Skin-only* cluster) are defined by several features that also define the unique genes of the prostate **(** **Table 1** **-** these categories are denoted by an asterisk). The important characteristic of the stem cell niche of providing physical contact [22] is indicated by the category *cell adhesion* (Table 1). In addition, many TF-binding motifs are shared between these two unique clusters. This suggests that even though each of these cell populations has a unique cohort of transcripts that defines almost half of its cellular signaling program, a common set of TFs may coordinate their individual transcriptional programs.

**Table 1:** Functional and promoter sequence motifs overrepresented within the Prostate-only cluster in the UGE and UGM compartments.

Functional category enriched in UGE	Gene Ontology ID	Genes associated with the category		Functional category enriched in UGM	Gene Ontology ID	Genes associated with the category	
anatomical structure development	0048856	128		anatomical structure development	0048856	136	
multicellular organismal development	0007275	136		multicellular organismal development	0007275	147	
cell adhesion *	0007155	51		cell adhesion *	0007155	51	
regulation of cellular process	0050794	192		ion channel activity	005216	49	
epidermis development	008544	21		extracellular space	000561	49	
intracellular signaling cascade *	0007242	78		cell soma	0043025	15	
				ion channel activity	005216	49	
				extracellular space	000561	49	
				cell soma	0043025	15	
				membrane fraction	0005624	43	
				calcium ion binding	0005509	63	
				cytoskeleton organization and biogenesis	0007010	40	
				cytoplasmic vesicle	00431410	25	

TF enriched in UGE	Accession number in TRANSFAC	p-value**	Enrichment factor ***	TF enriched in UGM	Accession number in TRANSFAC	p-value**	Enrichment factor ***
Ap2 *	M00915	2.2E-05	1.3	Ap2 *	M00189	3.5E-06	1.3
E2f *	M00803	8.0E-05	1.2	E2f *	M00803	3.8E-07	1.2
Lbp1 *	M00947	2.3E-07	1.4	Lbp1 *	M00644	8.4E-06	1.2
Sp1 *	M00196	2.8E-11	1.3	Zf5 *	M00716	2.0E-08	1.2
Areb6	M00414	2.5E-08	1.5	Lmo2 complex	M00277	1.5E-06	1.4
Hen1	M00058	1.8E-05	1.4	Nrsf	M00256	2.6E-06	1.4
				Maz *	M00649	2.3E-05	1.4
				Etf	M00695	2.3E-05	1.2
				TFII-I	M00706	3.6E-06	1.4

*Categories that are also enriched in the corresponding skin compartments.

**p values indicate the significance of TF signature enrichment in the gene set relative to that in the background set as described in Materials and Methods.

***Enrichment factor values represent the frequency of the TF signature in a cluster divided by its frequency in the background set.

Under the assumption that gene expression levels correlate with the level of their encoded proteins, the UGM compartment manifests a greater signaling complexity (indicated by its increased number of enriched functional categories) than the UGE compartment (**Tables 1**, **2**). This may reflect the role of the UGM in defining the appropriate signals that are essential for supporting and regulating the behavior of the primitive stem cells in their niche [7], [23]. It could also reflect the more heterogeneous cell population of the mesenchyme. A number of categories that are expressed in the *Prostate+Skin UGM/dermis* gene set (e.g. *organ morphogenesis*, *cell proliferation*, *response to external stimulus* and *cell migration*) (**Table 2**) may be involved in the supportive role the mesenchymal compartment plays in providing the appropriate microenvironment for the primitive epithelial compartments of the prostate and skin. A variety of functional categories such as *sialyltransferase activity* [24], *carbohydrate binding* [25], *receptor tyrosine kinase activity* [26] and *collagen* [27], [28] that are significant to the biology of stem cells are also enriched in the *Prostate+Skin UGM/dermis* gene set.

**Table 2:** Functional and promoter sequence motifs overrepresented within the Prostate+Skin cluster of UGE/epidermis and UGM/dermis.

Functional category enriched in UGE/epidermis	Gene Ontology ID	Genes associated with the category		Functional category enriched in UGM/dermis	Gene Ontology ID	Genes associated with the category	
plasma membrane	005886	50		plasma membrane	0005886	161	
anatomical structure development	0048856	70		proteinaceous extracellular matrix	0005578	84	
intracellular signaling cascade	0007242	46		multicellular organsimal development	0007275	243	
morphogenesis of epithelium	0002009	13		cell adhesion	0007155	114	
cytoskeleton	0005856	38		calcium ion binding	0005509	113	
kinase activity	0016301	40		cell migration	0016477	61	
phosphorus metabolic process	0006793	36		organ morphogenesis	0009887	78	
				blood vessel development	0001568	47	
				nervous system development	0007399	80	
				phosphate transport collagen	0005581	19	
				enzyme linked receptor protein signaling pathway	0007167	43	
				carbohydrate binding	0030246	42	
				regulation of cellular process	0050794	263	
				tube development	0035295	26	
				response to external stimulus	0009605	48	
				cytoskeletal protein binding	0008092	40	
				ion transport	0006811	64	
				negative regulation of biological process	0048519	75	
				transmembrane receptor protein tyrosine kinase activity	0005624	15	
				cell proliferation	0008283	45	
				sialytransferase activity	0008373	8	
				myofibril	0030016	15	
				intracellular signaling cascade	0007242	79	
				actin cytoskeleton	0015629	25	

TF enriched in UGE/epidermis	Accession number in TRANSFAC	p-value*	Enrichment factor **	TF enriched in UGM/dermis	Accession number in TRANSFAC	p-value*	Enrichment factor **
Sp1	M00196	1.1E-17	1.4	Sp1	M00196	7.8E-05	1.1
TFII-I	M00706	9.6E-05	1.4	TFII-I	M00706	3.5E-05	1.3
Srebp1	M00749	8.9E-05	1.4	Srebp1	M00749	4.5E-05	1.3
Lmo2 complex	M00277	7.1E-10	1.8	Mazr	M00491	5.0E-13	1.5
Areb6	M00414	8.9E-10	2.0	Maz	M00649	5.1E-12	1.4
Egr1	M00807	2.8E-07	1.4	Lbp1	M0644	4.4E-08	1.3
E2a	M00804	2.9E-05	1.5	Ap2	M00915	5.9E-07	1.3
				Rreb1	M00257	8.4E-07	1.3
				Spz1	M00446	4.6E-06	1.4
				Pax4	M00378	1.0E-05	1.3
				Lbp1	M00947	1.0E-05	1.3
				Zf5	M00716	1.9E-05	1.2
				Hen1	M00058	2.2E-05	1.3
				Smad	M00974	9.6E-05	1.4

*p values indicate the significance of TF signature enrichment in the gene set relative to that in the background set as described in Materials and Methods.

**Enrichment factor values represent the frequency of the TF signature in a cluster divided by its frequency in the background set.

The promoter sequence analysis indicates that some of the enriched binding motifs are shared by gene sets of the prostate and the skin, among them the binding site of TFII-I, Srebp1, Maz and Lmo2 (**Tables 1**, **2**). This indicates the central role that these TFs may play in the regulation of epithelial/mesenchymal niche interactions.

### C. The potential regulatory layers of gene expression in the prostate stem cell niche

Our analysis reveals that several TFs are potentially active in both the UGE and the UGM. Of interest is the finding that several TFs that may be active in the prostate stem cell niche may also be operative in the skin compartment. Ample evidence (see below) indicates that many of these TFs have central regulatory roles in controlling gene expression in other stem cell systems where they regulate functions such as self-renewal, migration and oncogenesis.

#### a) Self-renewal and maintenance of primitive cells

The enrichment of several TF-binding motifs in genes that are overexpressed in the prostate stem cell niche supports the notion that this is a self-renewing primitive compartment (**Table 3**). Expression of E2f (**Table 1**) is associated with the self-renewal of murine trophoblast stem cells [29], the expansion of stem cells in the columella and lateral root caps of Arabidopsis [30] and with poor prognosis in prostate cancer [31] Recently, genomic profiling indicated the functional role of the Rb-E2F axis in controlling quiescence in adult murine hair follicle bulge stem cells [32]. In addition, studies on impaired wound healing in E2F1-null mice, due to defective cell proliferation and thus reduced re-epithelialization, strengthen the role of E2F in the maintenance of proper stem cell self renewal in the epidermis [33]. Lastly, next-generation sequencing (ChIP-Seq) revealing binding patterns of twelve TFs that govern the regulation of differential gene expression in embryonic stem cells, has identified E2F1 as an activator that induces up-regulation of genes that allow maintenance of self-renewal and pluripotency [34].

**Table 3:** Transcription factors involved in the biology of the prostate stem cell niche.

Category	Transcription Factor
Self-renewal and maintenance of primitive cells	E2f
	Etf/Tead2
	Ap2
	TFII-I
	Smad
Lipid metabolism	Srebp1
Migration and tumorigenicity	Lmo2
	Egr1
	Areb6/Zeb1
	Rreb1
Neuronal lineage	Hen1/Nscl1/Nhlh1
	Nrsf/Rest

Etf/Tead2 (**Table 1**) promotes self-renewal of progenitor cells in the olfactory epithelium [35] and is an essential transcriptional regulator during the first week of murine embryo development [36]. In addition, Tead2 may be involved in the production or maintenance of embryonic stem cells as comprehensive transcriptional profiling shows that Tead2 is highly expressed in embryonic, neural and hematopoietic stem cells [37]. Since these analyses compared the transcript levels of proliferating stem cells relative to differentiated cells, the expression of Tead2 appears to be specific to proliferating cells suggesting a possible role in self-renewal of embryonic prostate stem cells.

**Figure 2:**
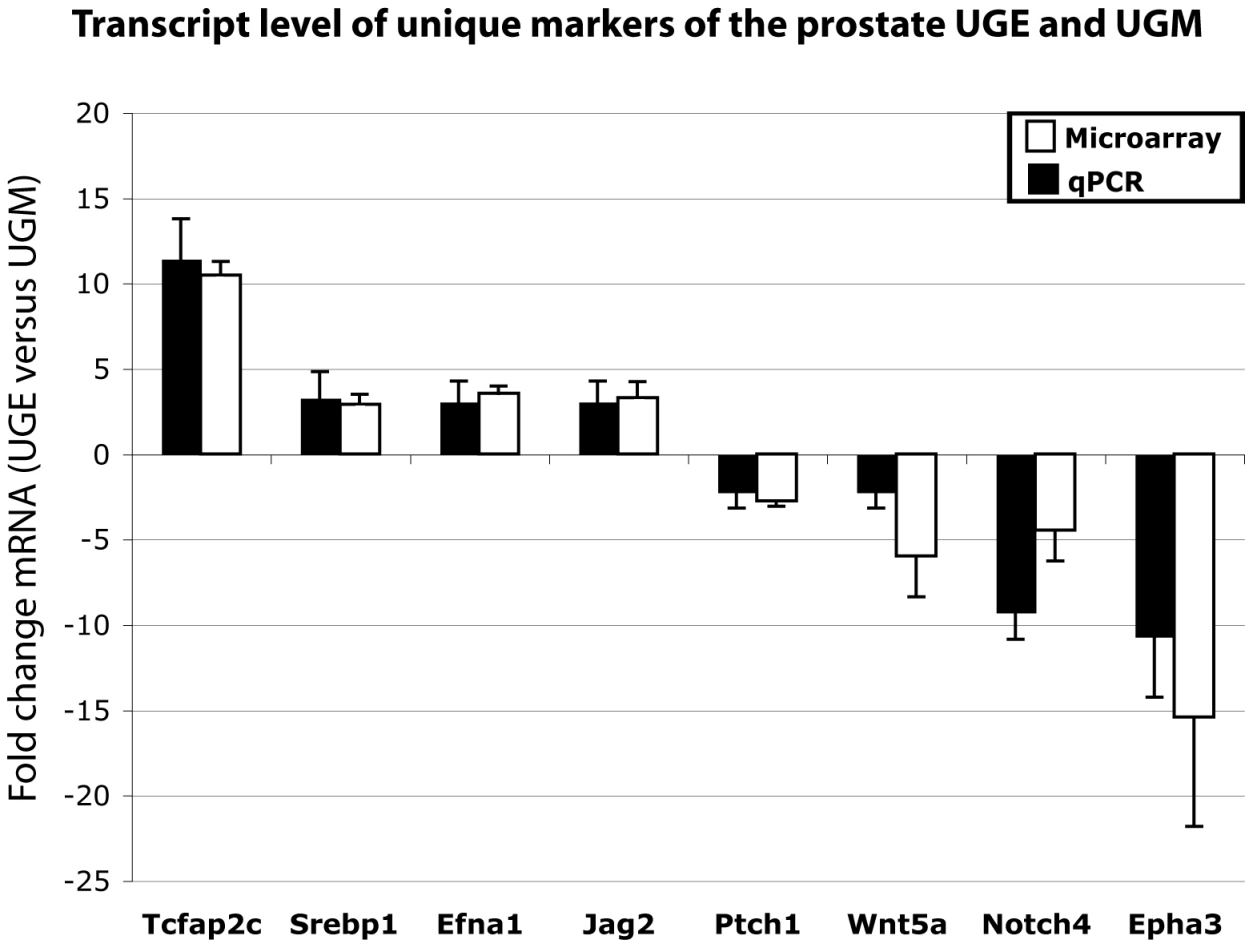
Transcript level of unique markers of the prostate UGE and UGM. Microarray data (white columns) of unique markers of the prostate niche are expressed as the fold change of mRNA levels of the UGE vs the UGM [mean±SD; n = 6]. Results of real-time quantitative PCR analysis (qPCR) (black columns) are presented as the fold change of transcripts after normalization of UGE vs UGM [mean±SD; n = 3].

Additional support for self-renewal in the prostate stem cell niche is indicated by Ap2 (**Table 1**) that promotes proliferation over differentiation and is a marker of stemness and pluripotency [38]. Ap2 regulates basal progenitor fate in a region- and layer-specific manner in the developing cortex as its deletion during development results in a specific reduction of upper layer neurons in the occipital cortex, leading to impaired function and enhanced plasticity of the adult visual cortex [39]. Recently, a ChIP-chip and transcriptome study of transcriptional networks testing TF-occupancy of genes involved in trophoblast stem cell self-renewal has shown that Ap2 along with four other genes occupies gene promoters involved in maintaining the stem cell phenotype and pluripotency [40]. We find that the transcript levels of three members of the Ap2 gene family are altered in the prostate stem cell niche. The mRNA levels of *Tcfap2a* and *Tcfap2c* are higher in the UGE compared with the UGM by 3- and 10-fold respectively, while transcript levels of *Tcfap2b* are 6-fold higher in the UGM (**Table S1**). qPCR analysis validated the expression of *Tcfap2c*, indicating that it is significantly elevated in the UGE (11-fold) relative to the UGM (**Figure 2**). The transcription factor TFII-I (**Table 1**) restricts the expression of β-globin to the adult stage of erythropoiesis thus maintaining the primitive phenotype of murine embryonic stem cells [41]. In addition, the enrichment of the Smad motifs are in agreement with the known regulatory role of the TGF-β pathway in maintaining the primitive phenotype of embryonic stem cells [42] and in promoting quiescence of adult prostate cells [43], [44]. In this regard, it is important to note that the TGF-β pathway plays an important role in the activity of TFII-I proteins. In MCF7 breast tumor cells TGF-β-dependent phosphorylation of TFII-I results in reduced transcriptional activation of Ccnd2, Ccnd3 and E2f2 genes [45]. However, the addition of TGF-β to mouse P19 embryonal carcinoma cells induces transcription of TFII-I [46].

#### b) Regulation of lipid metabolism

Several findings support the notion that lipid metabolism plays a major role in the prostate stem cell niche. The binding motif of the sterol regulatory element-binding factor 1 (Srebp1/Srebf1), an androgen-regulated TF with a role in the metabolism of lipids [47], is apparent in Prostate*+Skin UGE/epidermis* and *Prostate+Skin UGM/dermis* gene sets (**Tables 2**, **3**). In addition, *Srebp1* mRNA is up-regulated by 3-fold in UGE relative to the UGM (**Table S1**). Analysis by qPCR confirms that *Srebp1* is up-regulated by 3-fold in the UGE cells compared with the UGM cells (**Figure 2**). Recent studies indicate that lipids have an important role in the self-renewal of stem cells [48], [49]. A recent study has shown that embryonic stem cells are characterized by highly unsaturated lipid metabolites that are important in maintaining ‘chemical plasticity’ and ultimately in mediating pluripotency. Inhibition of a pro-oxidative cascade that oxidizes highly unsaturated lipids promotes the pluripotent state of embryonic stem cells and inhibits differentiation [50]. Srebp1 contributes to the androgen-independent survival and proliferation of prostate cancer cells [51]. In addition, prostate tumors have an innate capacity to synthesize androgen from cholesterol [52], indicating the relevance of lipid metabolism for both normal stem and prostate tumor cell biology. In agreement with this, we note the enrichment of the functional category *plasma membrane* in two of the *Prostate+Skin UGE/epidermis* and *Prostate+Skin UGM/dermis* gene sets (**Table 2**). Genes that are specifically involved in lipid metabolism such as *Anxa7* are up-regulated in the UGE (3-fold) while others, such as *PrnP*, *Syt1* and *Dgkb* are highly expressed (4, 8 and 8-fold, respectively; **Table S1**) in the UGM. In this regard, fatty acids regulate gene expression and significantly stimulate the growth of human prostate cancer PC-3 cells [53]. Furthermore, inhibition of fatty acid synthase parallel to reduction of endogenous lipid synthesis induced by the green tea component, Epigallocatechin-3-gallate, leads to selective induction of apoptosis in prostate cancer cells [54]. In addition, several genes related to phospholipid metabolism such *Tiam1*, *Mdr3/Abcb1a*, *Lgals* and *Dlk1*, *Pltp* and *Akp2* are up-regulated in both adult and fetal prostate stem cells [43]. The contribution of this group of genes to the stem cell niche may be especially significant because proteins active in signaling pathways of stem cells (e.g., Wnt proteins) are known to undergo lipid modification that regulates their ability to maintain stemness [55].

#### c) Promotion of migration and tumorigenicity

Several of the TF-binding motifs that are enriched in the prostate stem cell niche are implicated in the development of prostate cancer (**Table 3**). Binding motifs of the LIM domain only 2 (Lmo2) are enriched in *Prostate-only UGM* and the *Prostate+Skin UGE/epidermis* gene sets (**Tables 1**, **2**) and the transcript level of *Lmo2* is 11-fold higher in the UGM relative to the UGE (**Table S1**). In normal prostate, Lmo2, a regulator of cell fate and differentiation, was shown to be expressed exclusively in the basal cell layer [56]. In addition, overexpression of Lmo2 is significantly associated with the advanced stage of human prostate tumors and stable expression of LMO2 in LNCaP cells promotes cell motility and invasiveness *in vitro* [57]. Abundant evidence indicates that the early growth response-1 protein (Egr1) has a role in prostate cancer [58], [59], [60]. The increased activity of Egr1 (**Tables 2**, **3**) was recently found to promote the growth of prostate cancer cells in an androgen-depleted environment [61], suggesting that prostate tumor cells may adopt regulatory pathways expressed in prostate stem cells. In support of this finding, down-regulation of Egr-1 by siRNA inhibits PC-3 human prostate tumor growth and induces apoptosis [62]. In addition, E2F1 induces tumor cell survival and promotes resistance to drug-induced apoptosis via induction of Egr1 transcription in the human prostate cell lines DU145 and PC3 [63].

Enrichment of Areb6/Zeb1 motifs in both the *Prostate-only UGE* and the *Prostate+Skin UGE/epidermis* gene sets suggests that cell migration may be an integral component of the prostate stem cell niche. This TF is a regulator of epithelial-mesenchymal transition in prostate cancer and stimulates migration and invasion of prostate cancer cells [64]. Interestingly, epithelial-mesenchymal transition has been shown to generate cells with properties of stem cells [65]. The activity of Areb6 in the prostate stem cell niche may be related to this process in the primitive niche as well as to the invasion of the epithelial stem cells into their surrounding mesenchyme to form the primitive prostate buds from which the prostatic ducts develop [11]. Another regulator that may affect cell migration in the prostate stem cell niche is Rreb1 (**Table 3**) that suppresses AR-mediated promoter activity in prostate cancer cells [66]. In addition, Rreb1 is essential for diminishing the cell-cell adhesive interactions of epithelial cells and is required for their migration [67]. Thus, the combination of transcription factors expressed in the primitive prostate stem cell niche may dictate the dynamic changes in morphology and migration of cells within their niche.

#### d) Expression of neuronal lineage markers in the prostate stem cell niche

Our analysis also identifies cellular regulators that may be related to the neuronal lineage. The *Prostate-only UGE* and *Prostate+Skin UGM* gene sets are both enriched in the Hen1/Nscl1/Nhlh1 binding motif. Hen1 is a neural cell-specific TF that is expressed in cells in the developing pons in the embryonic murine brain. It orchestrates the migration of neuronal precursor cells and also inhibits apoptosis thus promoting their survival [68]. Another TF binding motif that is active in the neuronal lineage is Nrsf/Rest. This TF maintains self-renewal and pluripotency, preventing differentiation in embryonic [69] and neuronal stem cells [70]. In contrast to TFs that induce expression of their target genes, Nrsf is a transcription repressor and thus its repressive activity down regulates its target genes. Therefore, the enrichment of its binding motif in the UGM implies that these genes are relatively highly expressed in the UGM as a result of the suppressive activity of Nrsf in the UGE (**Table 1**). For example, the expression of *Synapsin1* transcripts, an Nrsf-suppressed target gene, is 7-fold lower in the UGE relative to the UGM (**Table S1**). Our functional analysis supports these observations as one of the categories that is enriched in the *Prostate+Skin UGM/dermis* gene set, *nervous system development*, is related to the neuronal lineage (**Table 2**). These data indicate that the neuronal and prostate stem cell niches may have a number of commonalities. Interestingly, the molecular signature of adult prostate stem cells indicates that it resembles that of neuronal or embryonal progenitors to a greater extent than that of hematopoietic progenitors indicating some commonalities with the neuronal lineage [43]. It is also possible that neuronal progenitor cells may be present within the UGM and dermal compartments.

### D. Ligand – receptor interactions in the prostate stem cell niche

One of the major cellular mechanisms by which stem cells convey signals within their niche is via ligand-receptor interactions. Both the identity of the specific ligand-receptor interaction as well as its quantitative nature have a significant effect on stem cell fate [71]. We therefore used our differential gene expression profiling data to obtain insights into the ligand-receptor interactions that may mediate signals between the UGE and UGM in the prostate stem cell niche. We identified two types of ligand-receptor interactions that, based on the differential gene expression profiles in UGE/UGM, could potentially be active in the embryonic prostate stem cell niche:

1. paracrine interactions in which the receptor is expressed by one of the compartments while the ligand is expressed by the corresponding compartment (e.g., UGE-ligand and UGM-receptor or vice versa) and
2. autocrine interactions in which both the ligand and the receptor are expressed by the same compartment (e.g., UGE-ligand and UGE-receptor) (**Table 4**).

**Table 4:** Ligand-receptor pairs whose transcripts are up-regulated in UGE and UGM.

Category	UGE ligand	UGM receptor	UGM ligand	UGE receptor	
Wnt signaling	Wnt4 (x23)*	Fzd1 (x4)	Wnt2 (x12)*	Fzd6 (x6)*	Paracrine and autocrine signaling
	Wnt6 (x6)	Lrp8 (x3)	Wnt5a (x6)*	Lrp2 (x4)	
	Wnt7b (x13)	Lrp11 (x11)	Wnt5b (x4)	Lrp4 (x2)	
	Wnt10a (x15)		Wnt9a (x5)*		
			Wnt11 (x8)		
Notch signaling	Jag2 (x4)	Notch4 (x4)*	Dll1 (x2)	Notch1 (x4)*	
			Dlk1 (x4)*	Notch3 (x4)	
Ephrin signaling	Efna1 (x3)	Epha3 (x9)*	Efna2 (x3)	Epha1 (x8)*	
		Epha4 (x5)		Epha2 (x4)	
		Epha5 (x12)			
		Epha7 (x4)			
		Ephb1 (x13)	Efnb1 (x2)	Ephb6 (x2)	
			Efnb3 (x6)*		
Fibroblast growth factor signaling	Fgf1 (x4)	Fgfr1 (x3)*	Fgf14 (x2)	Fgfr2 (x3)	
			Fgf13 (x4)*	Fgfr3 (x21)*	
			Fgf7 (x4)*		
Inhibin signaling	Inha (x2)*		Inhba (x16)	Acvr1b (x3)*	
				Acvr2b (x2)	
Bone morphogenetic protein family			Bmp4 (x12)*	Bmpr1b (x2)	
			Bmp5 (x5)*	Acvr2b (x2)	
			Bmp6 (x2)		
Hedgehog signaling	Shh (x34)	Ptch1 (x3)			
		Ptch2 (x4)			
Neurotrophic tyrosine kinase	Ntrf5 (x3)	Ntrk2 (x9)*	Ngfb (x2)		
		Ntrk3 (x8)	Ntf3 (x3)		
		Ngfr (x17)*			
Platelet-derived growth factor	Pdgfa (x6)	Pdgfra (x11)*			
Tgf-beta signaling		Tgfbr2 (x5)*	Tgfb2 (x5)*		Autocrine signaling
		Eng (x3)	Tgfb3 (x11)*		
		Ltbp1 (x2)			
Angiogenesis signaling		Tek (x7)*	Angpt1 (x14)*		
			Angptl1 (x7)*		
		Kdr (x14)*	Vegfc (x6)		
			Flgf (x4)*		
		Flt1 (x6)*	pgf (x4)		
		Nrp1 (x6)*			
Protein tyrosine phosphatase		Ptprz1 (x6)	Ptn (x4)*		
		Ptprb (x10)*			

*Ligand or receptor transcripts that are also overexpressed in the epidermis/dermis.

**Figure 3:**
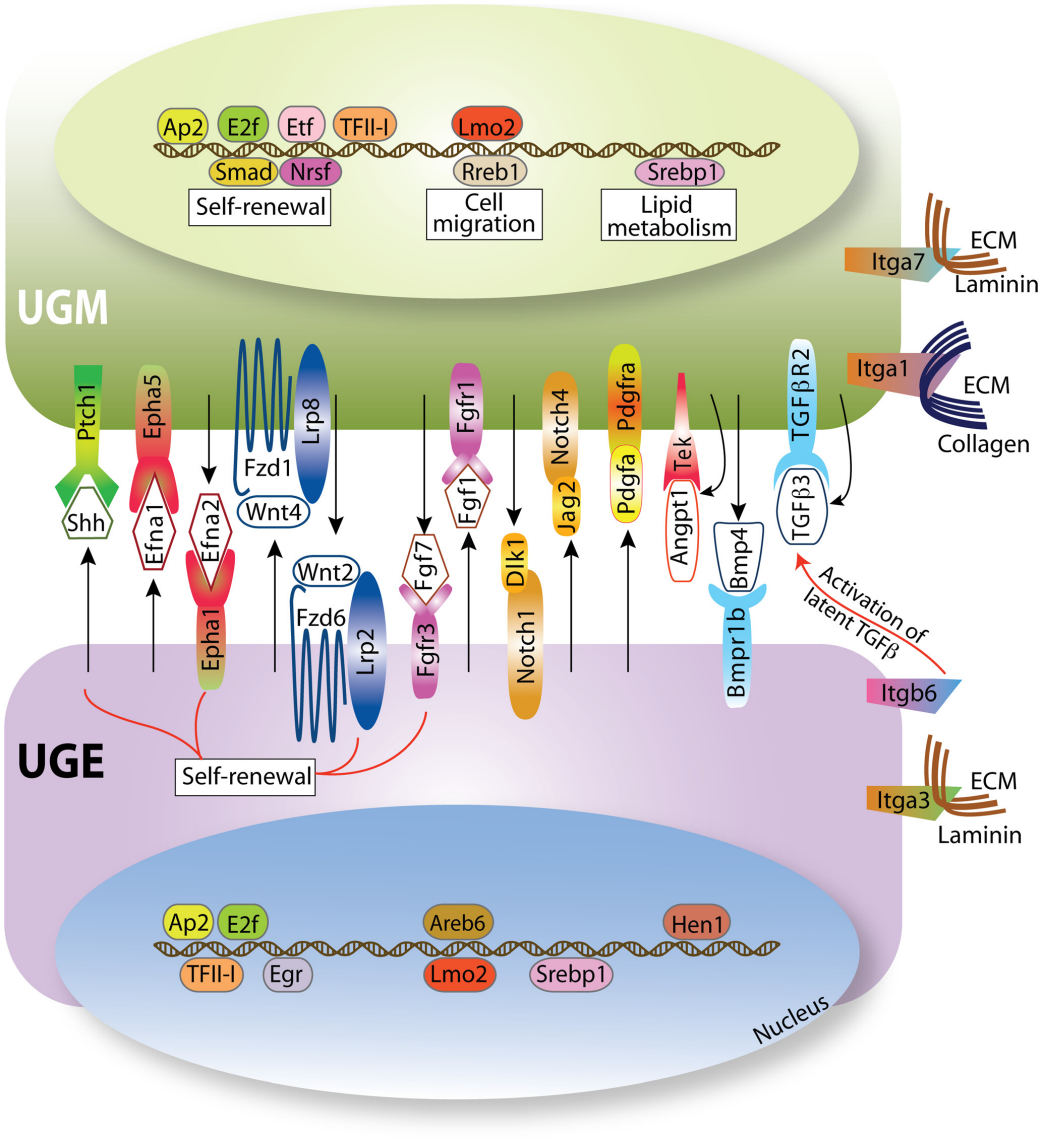
The signal transduction pathways expressed in the prostate stem cell niche. Ligand-receptor interactions and transcription factors that are differentially expressed between the UGE and the UGM are depicted. These are the main mediators of the cellular cross-talk between these two compartments in the prostate stem cell niche. These mediators include members of the Shh, Wnt/β-catenin, Notch, FGF and TGF-β signaling pathways that are important regulators of stem cell self-renewal and differentiation. Transcriptional regulators whose binding motifs are enriched in the differentially expressed genes are depicted in the nucleus. Activation of these transcription factors promotes expression of genes that are involved in self-renewal, lipid metabolism and cell migration. Many of these factors are implicated in the progression of prostate tumors as their expression is increased (e.g., Wnt/β-catenin genes) or decreased (e.g., Notch genes).

This survey indicates that some of the predicted ligand-receptor interactions identified in the prostate stem cell niche (**Figure 3**) are also present in the epidermis/dermis compartment (**Table 4**, indicated by an asterisk). The Wnt/β-catenin pathway that promotes self-renewal in various types of stem cells, such as keratinocytes, embryonal, colon, intestinal and follicular stem cells [72], has the greatest ligand-receptor representation in the prostate stem cell niche (**Table 4**). For example, *Wnt5a* expression is 6-fold higher in the UGM compared to the UGE (**Figure 2**). In the bone marrow niche this signaling pathway is essential for maintaining the quiescent state of hematopoietic stem cells [73]. Activating mutations and overexpression of numerous components of the Wnt/β-catenin pathway are present in patients with advanced prostate tumors [74], indicating that these tumors may arise in the primitive compartment. As mentioned above, lipid modification plays a major role in Wnt signaling, as these proteins require palmitoylation for their activity [55], indicating that lipid metabolism and the Wnt signaling pathway are both important regulators in the prostate stem cell niche.

The Wnt/β-catenin pathway also regulates the expression of members of the family of Eph receptors and their ephrin ligands [75]. Eph-ephrin interactions are responsible for positioning cells in the stem cell niche and promote proliferation of progenitor cells [76]. Several ligands and receptors of this family are highly expressed in the epithelial and in the mesenchymal compartments of the prostate stem cell niche (**Table 4**, **Figures 2**, **3**). Specifically, qPCR analysis indicates a 3-fold increase in the *Efna1* ligand in the UGE and a 11-fold increase in expression of the ephrin receptor *Epha3* in the UGM (**Figure 2**). Ephrin ligands and their receptors may have clinical relevance as their expression pattern differs between normal prostate epithelium and prostate carcinomas [77]. Notably, expression of two ephrin receptors that are present in the prostate stem cell niche, *Epha1* and *Epha3*, is decreased in prostate cancer compared with the normal tissue [77].

An additional ligand-receptor pair that is expressed in the prostate stem cell niche (**Table 4**) and interacts with the Wnt/β-catenin pathway is that of platelet derived growth factor (PDGF) and its receptor. Up-regulation of β-catenin, induced by PDGF, promotes the formation of a multipotent bone marrow-derived mesenchymal stem cell niche [78]. PDGF induces epithelial-mesenchymal transition in prostate cancer PC3 cells, promotes tumor growth and contributes to the “cancer stem-like” phenotype associated with epithelial-mesenchymal transition [79]. This ligand and its receptor may therefore be important regulators of stem cell behavior in the prostate stem cell niche.

Another major signaling pathway that is relevant to embryonic and postembryonic stem cell biology is the Notch pathway [80] that is represented in the prostate stem cell niche by several ligand-receptor candidates whose level is differentially expressed in one of the niche compartments (**Table 4**). For example, the UGE compartment expresses increased levels (4-fold) of the Notch ligand, *Jag2*, while the UGM cells express relatively high levels (4-fold) of another family ligand, *Notch4* (**Figures 2**, **3**). This pathway is important for the early development of the prostate and for branching morphogenesis [81], [82]. Inhibition of Notch signaling leads to dramatic alteration in prostatic morphogenesis, including increased budding and epithelial proliferation. In addition, expression of Notch1 is significantly lower in human prostate carcinoma compared to normal tissues [81], [82]. This indicates that abrogation of the inhibitory effect of Notch signaling in the niche may promote the expansion of primitive cells, thus facilitating evolution of prostate tumors.

Fibroblast growth factor (FGF) signaling has a major role in the self-renewal of human embryonic stem cells [83], [84] and is essential for the maintenance of their supportive niche [85]. Members of the FGF family also participate in mesenchymal/epithelial interactions during organogenesis and carcinogenesis. Impaired FGF signaling promotes epithelial-mesenchymal transition and tumor progression in murine prostate cancer models [86]. Fgfr2 is important for prostatic branching morphogenesis and for acquisition of androgen responsiveness in mice [87]. Our analysis for genes participating in ligand-receptor interactions reveals an abundance of FGF family members that may participate in the regulation of this pathway (**Table 4**, **Figure 3**). An important indication of the role of FGF signaling in the prostatic stem cell niche is the finding that enhanced mesenchymal expression of Fgf10 promotes multifocal intraepithelial neoplasia lesions in the prostate. Furthermore, inhibition of epithelial Fgfr1 signaling leads to reversal of the cancer phenotype, suggesting that stromal Fgf10 expression promotes the multifocal formation of prostate carcinoma [13].

TGF-β and BMPs, that are members of the TGF-β superfamily, also initiate pathways that are highly relevant in maintaining stem cell self-renewal [42], [88]. A number of these molecules are overexpressed in the UGE and UGM (**Table 4**, **Figure 3**). TGF-β signaling has a central role in mediating epithelial-mesenchymal interactions within the adult prostate stem cell niche [44]. The loss of the TGF-beta type II receptor gene in murine stroma results in intraepithelial neoplasia in prostate and an increased abundance of stromal cells [12], [89].

Other potential stem cell related ligand-receptor pairs that are identified by our survey in the prostate stem cell niche belong to the Shh-signaling pathway. This pathway induces proliferation of primitive human hematopoietic stem cells [90]. Shh-signaling is important for normal prostate growth and increases during development of prostate tumors that have increased expression of progenitor cell markers [91], [92], implying that primitive cells with an active Shh pathway may be expanded during tumorigenesis. The increased expression of the receptor for Shh, *Ptch1*, was confirmed by qPCR to be increased by more than 2-fold in the UGM compared to the UGE (**Figure 2**).

The Tek (Tie2)/angiopoietin1 signaling pathway that regulates hematopoietic stem cell quiescence in the bone marrow niche [93], is another pathway whose members are present in the prostate niche (**Table 4**). Plasma levels of the soluble Tek receptor are elevated in prostate cancer patients [94]. Expression of angiopoietin-2, the antagonist of angiopoietin-1, correlates with severity of disease in prostate cancer [95], suggesting that interference with the angiopoietin-1-Tek interaction may disrupt stem cell homeostasis.

### E. Integrin expression in the prostate stem cell niche

Integrins mediate attachment of stem cells to the matrix in the niche. Interaction of integrins with their matrix initiates signaling pathways and can also activate growth factor receptors [96]. In the UGM we find an increase of *Itga1* (4-fold), *Itga4* (13-fold), *Itga7* (2-fold), *Itga8* (3-fold), *Itga9* (4-fold) and *Itgb3* (2-fold). (**Table S1**).

A number of transcripts for other integrins implicated in stem cell biology and prostate cancer are up-regulated in the UGE. The α3 (*Itga3*) (up-regulated by 3-fold in the UGE) and β1 integrin subunits pair to form the α3β1 (VLA3) complex and the α6 and β4 (*Itgb4*) (up-regulated by 10-fold in the UGE) (**Table S1**) subunits pair to form the α6β4 complex, both of which are expressed in keratinocyte stem cells [96] and function as receptors for laminin, a major component of the basement membrane. The expression of the α3 integrin subunit is significantly increased by p63 [97]. Expression of *Trp63* is up-regulated 32-fold in the UGE relative to the UGM (**Table S1**). This may be responsible for the elevated expression of the transcript for the α3 integrin subunit in the UGE [97] which may influence the attachment of prostate stem cells to their niche. Interestingly, the transcript for integrin β6, an epithelial specific integrin [98] that is up-regulated 5-fold in the UGE, (**Table S1**) is involved in the activation of latent TGF-β3 [99] as well as TGF-β1 [100]. TGF-βs are secreted as latent molecules and require activation prior to binding their signaling receptor and the generation of active cytokine is one of the major regulators of the TGF-β signaling pathway. The absence of β6 integrin recapitulates the phenotype of TGF-β1-null mice indicating that this integrin is crucial for regulating TGF-β activity [100]. We have previously shown that the stem cell enriched proximal region of prostatic ducts has high levels of active TGF-β and TGF-β signaling promotes quiescence of stem cells in this region [44]. This signaling pathway is therefore likely to be an important regulator of the prostatic stem cell niche.

### Conclusions

These data provide, for the first time, a comprehensive description of the transcripts that are differentially expressed in the embryonic UGE compared to the UGM and reflect the molecular interactions that are likely to occur in the embryonic prostate stem cell niche. To our knowledge this is the first characterization of the potential signaling pathways and receptor/ligand interactions in epithelial and mesenchymal compartments isolated from a mammalian stem cell niche. As depicted in our model shown in **Figure 3**, the differential gene expression of specific signaling molecules in the UGE and the UGM may potentially result in expression of protein ligand-recptor pairs that mediate signal transductions in the embryonic prostate stem cell niche. For example, self-renewal of stem cells in the embryonic niche may be accomplished by paracrine interactions resulting from the expression and secretion of Shh by the UGE and its binding to the Ptch1 receptor, expressed by the UGM (**Figure 3**). In this regard, it is important to note that some of the postulated ligand-receptor interactions that we are proposing in the prostate stem cell niche could also be shared by other primitive niches, as we find similar differential expression of a number of these genes in embryonic skin (**Table 4**). For example, some of the Wnt pathway genes, such as Fzd6, Wnt2 and Wnt4, are also differentially expressed by the embryonic dermis and epidermis. There are however genes belonging to signaling pathways such as Ephrin and Shh whose differential expression is confined to the prostate stem cell niche (**Table 4**).

As we are aware of no transcriptional or protein expression data from other mammalian stem cell niches, a comparison between the prostate stem cell niche and other primitive niches can not be made. However, we expect that the detailed transcriptional profiles of the epithelial and the mesenchymal compartments of the embryonic prostate stem cell niche will be a useful source of data for such comparisons in the future. We should note, however, that some of the gene expression differences described in this study may be associated with more general epithelial-mesenchymal interactions rather than specific niche interactions. Future studies will determine protein expression by differentially expressed transcripts in the embryonic prostate stem cell niche. The expression of interesting proteins will be examined during fetal prostate development as well as in the adult prostate. As stem cells in the adult prostate reside in the proximal regions of ducts the proximal–distal prostate axis will be examined for evidence of differentially expressed molecules. This would indicate similarities between the adult and the fetal stem cell niches. As many of the interactions that regulate growth and differentiation in the normal adult prostate stem cell niche may also be involved in the aberrant proliferation of niche cells, their elucidation may provide insight into mechanisms subverted during abnormal proliferative events that result in benign prostate hyperplasia and prostate carcinoma. Importantly, it has been noted that prostate tumors originate in the stem cell enriched proximal region of prostatic ducts [9].

## Supporting Information

Table S1 Transcripts significantly expressed in the prostate subset. Lists of transcripts that were significantly highly expressed in the UGE compare to the UGM. The relative expression values (LR) compared to the average expression values of UGM samples are presented.
(4.81 MB XLS)

Table S2 Transcripts significantly expressed in the skin subset. Lists of transcripts that were significantly highly expressed in the epidermis compare to the dermis. The relative expression values (LR) compared to the average expression values of dermis samples are presented.
(1.26 MB XLS)

Table S3 Transcripts significantly expressed in the prostate and the skin niches. Lists of transcripts that were significantly highly expressed in each of the gene sets (Prostate-only UGE, Prostate-only UGM, Prostate+Skin UGE,Epidermis, Prost+Skin UGM,dermis, Skin-only epidermis, Skin-only dermis). Relative expression values (LR) are presented.
(3.51 MB XLS)

Table S4 Functional analysis of prostate gene sets. A list of the genes associated with each of the enriched functional categories that were identified for each of the gene sets.
(0.32 MB XLS)

Table S5 Promoter sequence analysis of prostate gene sets. A list of the genes associated with each of the enriched promoter motifs that were identified for the prostate gene sets.
(0.67 MB XLS)
